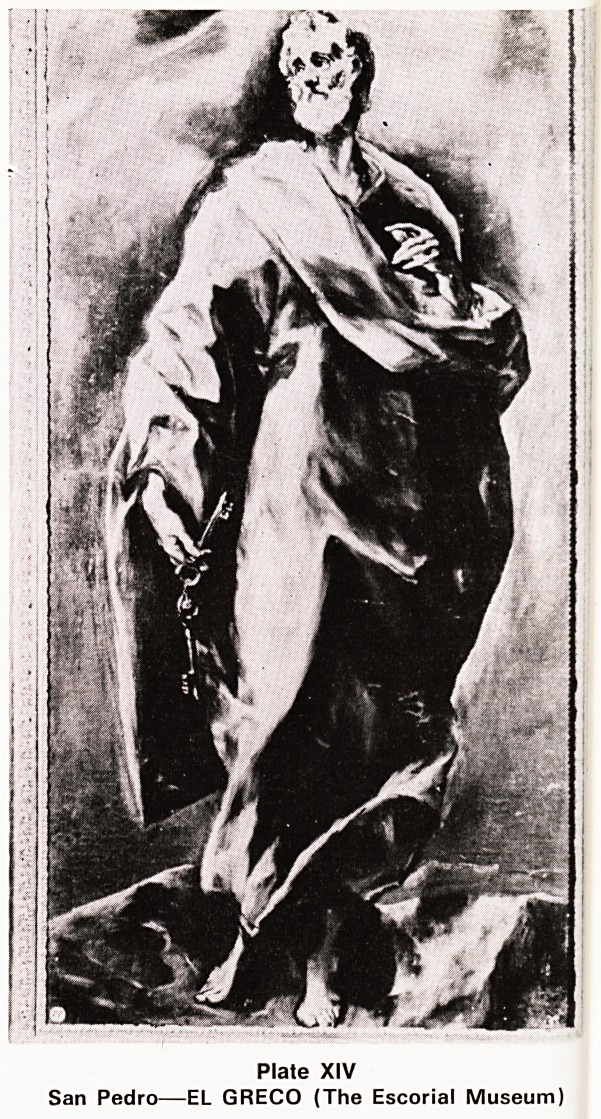# The Perfectionist
*Presidential Address to Bristol Medico-Chirurgical Society, October 1971.


**Published:** 1973-01

**Authors:** C. C. Morgans


### The Perfectionist*

C. C. Morgans, M.A., M.B., B.Chir.

When you elected me as your President you drew attention to my efforts to draw together doctors and ministers of religion for Discussion and Friendship. You will forgive me if I choose a subject from this sphere.

Sir Malcolm Sargent once said (Reid, 1968): "Sometimes I regret that I haven't given more time to specialising in certain composers. But then I tell myself that *somebody* has to be a General Practitioner of music and it looks as if that is to be my lot."

This can be said of so many of us in the Medical field, and I myself have enjoyed thirty-five interesting years of General Practice. In these days of Specialisation somebody has to hold together all the aspects of a case and treat a patient as a "whole man". In this way the G.P. is really a central figure in the National Health Service and *his* speciality is to be a really good "Jack-of-all-trades" as far as his ability reaches. I welcome, therefore, the upsurge of postgraduate training for the G.P. It is necessary for him to retain his ideals and to be as much of a perfectionist in his work as he possibly can. It is good to see that the best products of our Medical Schools do indeed emerge with these high ideals of perfection in their work. Dull indeed is the young Doctor who has not something of this starry-eyed enthusiasm and it is sad if he loses this in the struggle of life. At the same time, it is possible to set one's standards too high and to have them pulled down peg by peg by the pressures of life in General Practice. I am not at all thinking of the exaggerated perfectionism of the obsessional neurotic. There is, I think, something of the perfectionist in all of us who have been gripped from childhood with religious ideals. For some of us, to hang a picture requires finding the exact centre of the space available by measurement; to fold a letter means to make the corners meet exactly; if asked the time we must give it to the nearest minute; and butter and marmalade must be equally divided to suit the area of toast available! Garden plants must be arranged at regular intervals and in orderly rows, and tea-making for such a person becomes a matter of careful routine, involving measurement of tea and water and the warming of both teapot and hot water jug. One man I know even warms the cups as well! Such a person thinks of something he must remember to do and cannot rest until he has made a note of it or actually done it.

The perfectionist woman knows at once if anyone has been meddling, for all her cups and saucers and cutlery are piled tidily in the same places. She is greatly troubled by dust and spots and marks and dirty shoes bringing in unwanted mud. She is careful with her recipes and always reads the instructions on her tins before using them. She finds it difficult to trust other people to do things as she wants them done, and her motto is, "if you want a thing done well, do it yourself". She cannot bear to be in the wrong and must have the last word at all costs.

The perfectionist is marked by a certain rigidity, for he is tied to the wheel of rule and order and can only be partially saved by a sense of humour. Intense perfectionists border on being obsessional neurotics and are exasperating to live with and real bores. One woman said that her reaction to this trait in her husband was that she wanted to break everything in the house! Gwen Raverat writes about her perfectionist Aunt Ida, who was a member of the great Darwin family (Raverat, 1952), "her artist's nature was always driving her on to aim at perfection in everything, and so she could never herself obtain satisfaction. In manners and in morals; in rising up and lying down; in the roasting of the chicken and the trimming of Nora's garden hat; everything had to be perfectly done in the only right way. This made life difficult at times; a little slackness is a comfortable thing!"

It is necessary to say, however, that perfectionism has its uses. Mr. Michael Riley wrote recently (Reilly, 1971) that "the only defence for a surgeon against mistakes is to train oneself in a meticulous technique which reduces the possibility of mistakes to the minimum". Sir Kenneth Clark said (Clark, 1969) that "he liked the idea of Mozart sitting at table absentmindedly folding and refolding his napkin into more and more elaborate patterns as fresh musical ideas passed through his mind, a sort of formal perfection by which he used to express a passionate interest in human beings."

The Institute of Advanced Motorists believes that part of the answer to safety on the road is to teach a system of driving to which all drivers adhere, thereby cutting out as far as possible the "unpredictables"; and that the other part of the answer is a meticulous care of tyres and plugs and mirrors; in other words, every driver a perfectionist! It was once reported in the press that Lady Beaverbrook owed her life to the meticulous nature of her chauffeur, who bent down to polish the wheels, and caught sight of a bomb attached below the chassis (few of us, who are fads, are rewarded in this way!).

It is perhaps true that in this scientific age of computers and machines and sophisticated equipment nothing can quite replace the old-fashioned virtues of consistency and the refusal to "pass the buck".

The practice of medicine is a consistent fight to live up to ideals, keep up to date, improve one's techniques, differentiate between real progress and changing fashions. The young conscientious doctor should be encouraged not to give up his ideals. The perfectionist has a hard time, but it is worth it; and it does make the night call a shade easier if, when half asleep and in the half light, your clothes are placed in the reverse order for dressing quickly!

But Perfectionism has also disadvantages.

I find what is now, I suppose, an old-fashioned concept of the personality, nevertheless rather useful;

*Presidential Address to Bristol Medico-Chirurgical Society, October 1971.

namely the ego or natural self and the super ego or moral self, the two being so often in conflict. Hadfield (Hadfield, 1950) describes it as "the basic conflict in all the psychoneuroses"; the "nervous breakdown being the refusal of the natural self to conform any longer to the too rigid demands of the super ego", built up by the morals of society, by experiences, threats, punishments and teaching of parents, schools, social standards, and so on.

The Perfectionist becomes overloaded; he cannot "attain", or ever achieve even the standards he ha[illegible] set himself, and life becomes a burden.

J. B. Phillips says (Phillips 1967), "Perfectioni[illegible] obsession can make us arrogantly critical of oth[illegible] people, and in certain moods, desperately critical [illegible] ourselves — the Tyrannical 'Super-Me' condemns an[illegible] has no mercy on 'myself'." Moreover, his pride an[illegible] conceit may make him a "pain in the neck" to wor[illegible] with, in spite of his usefulness in a department. S[illegible] Kenneth Clark (Clark, 1969) talking of the symmet[illegible] of an Adams mantelpiece, remarks that "consis[illegible]tency and symmetry are enemies of movemen[illegible] consistency is enclosed, it becomes the prison [illegible] the spirit". Sooner or later the Perfectionist is broug[illegible] down, and will have to lower his sights. The "greatest[illegible] Cassius Clay meets someone more powerful; Dave Be[illegible]ford with his tremendous standards, falters and fal[illegible] out. Georges Simenon, the creator of Maigret, on[illegible] said, "I have tried to build here a kind of Perfection[illegible] have not achieved it; I cannot find inward peace". [illegible] Tony Hancock, the brilliant comedian, it was sai[illegible] "Throughout his career he drove himself too hard [illegible] search of Perfection"; and of Michelangelo, "All [illegible]

**Plate I—"The Unknown Political Prisoner"**

**Plate II—"Perfect Proportions"**

life his sense of duty constricted him like a ring the wearer has outgrown. The total sum of these duties often exceeded his physical and moral strength" (Schott, 1963).

Occasionally a man may enter a feminine field like flower decoration, with its strict principles of size and colour and height and line of flowers used; the choice of container and background; the hiding of all stalks and wire. Imagine such a one presented with a theme which he has to express and being written off with such comments as "You have a good idea but please *do* cover your mechanics", or, "Ignorance of your subject has led to the use of unsuitable material"!

In imagination I picture myself entering for such a Perfectionist competition. The subject given was "The unknown Political Prisoner". I felt it had to be solitary, with evidence of imprisonment. The result is shown in Plate I, and it simply produced the curt comment, "Your wire is showing"! A second attempt to meet the subject "Perfect Proportions" is shown in Plate II, calling for vital measurements of a Beauty Queen. It produced only the cryptic comment, "I should say this represents Disproportion"!! Stung by these crude comments, in my fantasy I gave up flower decoration!

**Plate III—"Do your Best!"**

Even so have many people given up their quest for perfection. Plate III illustrates the story of the farmer who wished to increase his egg yield and so he inserted an ostrich egg into the hen's nest with the legend, "Do your best", as an encouragement to greater efforts for Perfection.

This is the story of many a religious Perfectionist, for the ideals sought are extremely high, and seem quite out of reach, so that many break down because of their inability to come anywhere near the standards of moral behaviour set before them.

Victor Gollancz (1957) describes the intolerable burden of the life of Law for the strict orthodox Jew; and as for the Christian he has before him a pattern of Perfection, not only of a wonderful life and a fascinating person, but of superb teaching of a very high standard, setting up as goals virtues like purity, integrity, justice and truth, a "Holiness" which, taken at its face value, seems frankly impossible. If such demands are made on an ordinary man you can be sure one of two things will happen; either he will put them aside with a sigh and reluctantly, or even cheerfully, settle for a less exacting goal; or he will struggle and strive and go from one failure to another, developing an ever-growing sense of guilt, a burden which the happy-go-lucky pagan knows nothing about, until nervous symptoms begin to appear, and the psychosomatic process proceeds, until he cracks in one way or another. It is frustrating and cruel to hold out holiness like a never attainable carrot before any poor ass who tries to reach it!

I must at once say that such obsessional Perfectionism is not a true description of the Christian Life which in reality has a note of freedom and joy which is unique. But where does the solution lie? It is insufficient to say "Perfection is not demanded of us; only that we attempt it"; or "Nothing is perfect in this world so just do your best with available resources". Perhaps a solution may be found if we study briefly and impartially the life of the first Christian Man, not a mythological character but a real person, about whom we know a great deal. The man is Symeon or Simon, later to be called Peter. It is possible to trace his life as Disciple, Apostle and Martyr using ancient manuscripts which have been tested and sifted by modern scholarship and supported by archaeological findings and by strong traditions handed down.

This man was not at all the fussy Perfectionist, but he was one who had a high opinion of himself and especially of his courage; who set his sights high and who crashed spectacularly, and was subsequently built up into a powerful rock-like character.

He was, of course, a Jew, living in Palestine in the town of Bethsaida, the fisherman's town on the east bank of the Lake of Galilee, a man of humble origin, and a fisherman, a member of a small syndicate of five who owned a boat. Later he lived at Capernaum and was a married man (with a mother-in-law). It seems that parts of the shore of the lake at that time were a crowded, built-up area. It is said that in between the hills and the lake a large industrial population was crowded on a narrow strip of land varying from 4½—2½ miles wide, and 10 miles long. This strip was divided into two almost equal parts by the town of Tiberias. Josephus estimated the population of Galilee as 3 millions at that time. At least half that number must have lived by the Western lake in a district about the size of Manchester and Salford. The lakeside must have been one continuous line of buildings with milling crowds of people and very little privacy. But there was the lake, and Simon knew this like the palm of his hand, all 13 x 8 miles of it. Lying in a trench 582 feet below sea level, it was, and is, fed by the river Jordan from the North and has its outflow to the Dead Sea in the South; sweet water full of fish, 156 feet deep at its deepest point, often sparkling and blue, but subject to treacherous weather and sudden squalls and storms. The Rabbis said that "Jehovah created seven seas, but the sea of Gennesaret was his delight".

Simon would know where the best shoals moved and sometimes their black fins were visible like rain on the water. They could, on occasions break nets, and boats could sink.

Fishing was done either with a seine or drag net with floats and weights; or with a cast net like a weighted bag drawn in by a rope; or sometimes by hook and line. In a very hot area fishermen often worked naked, though clothing was always at hand for coming ashore, as nakedness was considered shameful. Fishing was often done by night with oil flares and beating on metal pans to drive the fish towards the net. The boat would take four to six men, with a single sail and a little deck shelter rather like a small edition of the St. Ives fishing boats.

Simon was an uneducated man in the sense that he was not studied by Jewish or Greek standards, but he learned Greek as a second language. He was, however, an intensely inquisitive man, always asking questions. He was not a humble man; he was very sure of the rightness of his own convictions and with a high opinion of his own abilities and especially his courage. He was impulsive to a degree and hot tempered, quite ready to whip out a sword and have a slash. He was therefore a born leader and as he was a Galilean with a strong accent he probably belonged to the Zealot party, a sort of local I.R.A., hating the Roman occupation and ready to follow any powerful leader who would drive out the Romans, indeed brought up to believe that such a Messiah would rise up amongst them somewhere. In the East the rules of hospitality, the extended families, and the simple living standards made it possible for young men to break away for a time and follow a Holy Man who might possibly be that leader. For a time Simon followed the gaunt fanatical figure of John the Baptizer, but one day while attending to his nets his brother came to him with the news that he and John Baptist had found a new leader, one Jesus of Nazareth, the very man to lead them to victory.

It was not long before he met this Rabbi face to face and to his surprise he welcomed him with, "I hear you are Simon son of Jona (The Dove); I'll nickname you Cephas (in Aramaic) or Petros (in Greek) or Peter the Rock"; and so Simon Peter he became.

Whether this welcome was good for him is questionable. This strong, impulsive, hot-tempered man ma well have known inwardly that there were weak spot in his character, cracks in the armour of his courag eous talk, traits which might undermine the hig standard of bravery which was his opinion of himse and his moral capabilities; anyway, this great boost t his ego was given to him.

However that may be, Simon Peter was called awa from his fishing to follow for three years a master wh drew him with a wonderful fascination and promise a new kind of fishing, namely to capture the ears an hearts of men. Simon thought that he had so muc to give, but found that he had everything to learn; an so began amazing experiences, of which I can on refer to a few.

**The Disciple**

He was quickly conscious that this Rabbi had diffe ent ideas and standards; and other dimensions ke breaking through; mystical and miraculous events o curred which struck awe into the fisherman's hea yet always the leader was lovable as a friend and o to be followed to the death. It was impossible regard him as ordinary, the supernatural kept brea ing through. Some of these events no longer see miraculous to us, but others still do.

The Holy Man takes them up to a cloud-capp mountain for a while. Whether it was the bright su shine through the cloud, or a peculiar radiance in t face of the leader, but it seemed to shine and h clothing became dazzling. Simon had fallen asleep b woke to the amazing scene of recognisable forms great men apparently talking to him. He was terrifi and lost his head and made foolish suggestions, a then the shadow came again and the voice—"This my chosen son: listen to him!" This scene the fish man kept to himself, for no one surely would belie him, and many would not today.

On another occasion there was an experience on t lake. The master had already given some indicati that nature and his personality were linked, and p sonality at its highest may well have insights i nature we do not yet understand—speaking to a calming the elements, and spotting the presence

**Plate IV—The Calling of the Apostles—DUCCIO (from the National Gallery of Art, Washington D.C.)**

**Plate V—The Miraculous Draught of Fishes—RAPHA (Victoria and Albert Museum)**

fish. Meeting the master after a night of fruitless fishing they are ordered to put out into deep water. "If you say so I will", says the weary Peter and then there is a huge catch, the nets breaking, and the boats loaded and Peter is quite overcome and falls at the feet of this man — "Go away and leave me, sinner that I am". (Plate V)

On yet another occasion during a night on the lake there was a terrific storm and all hands battling against a head wind and a rough sea. Suddenly a ghostly figure appears walking on the water, recognisably the master, and at once reassuring — "Don't be afraid, it is I". Then Peter's bravado and his courage overflowed, "If it's you tell me to come to you on the water — can't I do what you do?" and so he walked; but failing to keep his eyes on the goal, he looked round at the howling gale and he was afraid and began to sink; but a hand is stretched out, "Why hesitate? How little faith you have." On this occasion all of them fell at his feet, "Truly you are the son of God".

I have said he was a man of curiosity and always asking questions:—

"How often do I have to go on forgiving people?"

"Is this parable for us or for everyone?"

"We have left everything and followed you, what shall we get?"

"Why do you wash my feet?"

"What will happen to this man John?"

But on one occasion *he* was questioned as leader of the group at a crucial moment for the master. "Who do men say I am? And who do you say I am?" and Peter was suddenly inspired. "You are the Messiah—

**Plate VI—Entrega de las Llaves a San Pedro—CATEMA (Musco del Prado, Madrid)**

the son of the living God". This ecstatic utterance was met with "Simon, son of the fluttering dove, you are favoured indeed; you are Peter the Rock and on this rock I'll build by Ecclesia. I'll give you the keys of the kingdom of Heaven." (Plate VI)

This great declaration was followed by a forecast of trial and passion and shameful death and amazing statements of resurrection which Peter passed over and forgot. But the idea of crucifixion for a triumphant Messiah he could not swallow, and he took the master by the arm, "never this for you—no, no". And after all the praise came the stern rebuke—"Satan get behind out of my way!"

So we come to the climax of these astounding three years. Still Peter had not understood what kind of a Messiah, what kind of a God. He clung to the idea that he would deliver Israel by his miraculous powers; and then the crash came.

Peter and John are chosen to prepare an upper room for the final meal and here the master, wrapping a towel round himself like a slave, began washing their

**Plate VII**
**Christ washing Peter's Feet—FORD MADDOX BROWN (Tate Gallery)**

dusty feet. He comes to Peter, "Not mine, never". "If I don't, you aren't with me." "Alright, my feet, hands and head, all." "No, once you are bathed you just need the dust of everyday washed off your feet." (Plate VII)

And then to a garden where he witnessed an intensity of suffering by the master, but only for a while, as Peter was quite unable to stay awake. Suddenly the place is alive with torches and soldiers. and out comes Peter's sword; but the master is arrested and terror catches at his heart and he is away with the rest — "all of them deserted and fled".

This desertion had been foreseen by the Master and Peter had revealed his high opinion of himself and his courage—"If everyone else falls away I never will"; and the strange rejoinder. "Before the cock crow, you will disown me three times".

But his courage had not all gone, and there was no bolt-hole for him. Helped by his friend John he gets into the courtyard where there is a fire and he can keep warm and perhaps catch a glimpse of the Master as he comes away from his trial. A maid suddenly challenges him, "Woman I do not know Him". Others ask him, "You are his disciple?" "I am not"; and a relation of the man he served, "Your accent is Galilean, didn't I see you with him?" Reinforced by curses and an oath, "Man you did not see me—I don't know what you are talking about; I don't know this man." And then the early dawn and the loud crowing of a cock.

At this moment all eyes were turned on the prisoner as he emerged despoiled, mock-robed, buffeted and gobbed on, but his eyes were on one person only, Simon Peter, and one wonders how to describe the look he gave him; offended? reproachful? I told you so? indignant? I think not; something which conveyed the understanding and compassion of a real friend, for its effect on Peter was to make him burst into tears and turn away and stumble out into the darkness. Bach in the St. Matthew Passion conveys something of his feeling in the long drawn-out phrase for the words "he wept bitterly".

**Plate VIII—The Repentant Peter—EL GRECO (The Phillips Collection, Washington D.C.)**

Peter was brought low—very low: really shamed and humbled to the dust. He knew now the crack in his armour, like millions of us since; his protestations of idealism, of courage above that of other men, had failed; not for him again the proud perfectionist line; he was really humbled. (Plate VIII).

Yet *he* did not commit suicide. We shall never know what he did, wandering about alone, blinded by his tears; skulking in the back streets of Jerusalem, or hiding amongst the crowds on the Via Dolorosa, or watching from a good way off as his friend was hammered to a felon's cross. But two days later he was with his friends again—perhaps in an upper room, hiding for fear of the Jews, still the leader of a band which had all let the side down.

However, on the morning of the third day things began to happen. Women came hurrying with news of an open and empty tomb with a huge stone rolled aside, and he and John ran to see for themselves. John, perhaps a younger man, outstripped him, and looking in, a flood of memories came to him to convince him of the greatest miracle of all. Peter, perhaps more heavily built, comes up and goes right in, and sees the curiously tidy appearance of the grave clothes, as if laid aside. (Plate IX).

At some point a message reached Peter, "the Master is risen and is going on ahead to Galilee. Tell the Disciples *and Peter* that he will meet them there."

Back in the old lakeside haunts, what more natural than that he should pick up the fishing again, when

**Plate IX—And They ran Together—BURMAND**

everything had collapsed and uncertainty reigned; b[illegible] another fruitless night follows and in the morning [illegible] man is seen on the beach and a voice calls out, "Sho[illegible] your net to starboard if you want a catch". John cal[illegible] out to Peter, "it is the Lord" and as they grapple wi[illegible] the huge catch, Peter, impulsive as ever, grabs h[illegible] clothing and plunges into the sea, swimming or wa[illegible] ing ashore. There, perhaps laid out on a rock, was [illegible] fire and fish cooking, and bread, and a welcome fro[illegible] one who seemed the same Master yet must be diffe[illegible] ent also; and after the meal (as Isaak Walton used [illegible] say, "Fish is best cooked on the bank immediate[illegible] after being caught.") Peter is taken aside, and for h[illegible] three denials he is required to make three affirmatio[illegible] of love and is given each time a commission to act [illegible] shepherd to the new flock of followers of his Maste[illegible] The healing words and looks were those of forgiv[illegible] ness and abiding friendship and love such as can on[illegible] be received by truly humble people.

### The Apostle

Many meetings occurred after this over many wee[illegible] until the disciples were quite sure of the reality of t[illegible] living person of their Master and were not at all di[illegible] mayed when the final parting came.

They closed their ranks, filled up the vacant pla[illegible] left by Judas, and Peter was the obvious leader. Th[illegible] were now "Apostles", "men who had kept compa[illegible] with Jesus and witnessed his personal resurrection[illegible] They awaited a promised influx of spiritual power a[illegible] they got it; mystical again, mixed up with wind a[illegible] fire and a mixture of languages so that the onlooke[illegible] accused them of inebriation. At this point Peter stan[illegible] up boldly and makes his first great sermon, fearless[illegible] accusing the Jews, both common people and leade[illegible] of murdering their Messiah, the Son of God. He is [illegible] changed man, humiliated beyond words, forgiven a[illegible] reinstated beyond belief, understanding at last t[illegible] nature of the whole business, and conscious of ne[illegible] powers and new courage. From now he lived by [illegible] strength not his own.

The new power is soon evident. Faced with a pa[illegible] lysed beggar at the Beautiful Gate, the Golden Ga[illegible] of Jerusalem, a man who was lame from birth, whini[illegible]

and begging for a gift, Peter says, "Silver and gold we do not have but somthing else we have got, the power of Jesus of Nazareth, stand up and walk"; and he did, and we are told that his ankle joints were strengthened sufficiently to allow him to leap and to jump and to walk. And so the work of healing began and there were places where sick folk lay about as in Solomon's cloisters, and they said that even if Peter's shadow fell on them they felt better.

The early church soon ran into major problems, Jewish Christians wanting to impose the burden of orthodox Jewish Law on those outsiders who became Christians, particularly with regard to eating foods which they considered unclean. Peter is at Caesarea at the house of one Simon a Tanner, and he is on the flat roof and drops off to sleep. Here he has a vision of a great sailcloth lowered down full of so-called unclean foods and is told to eat. He protests, and is convinced of the rightness of the laying aside of the prohibitions, and so slowly the battle for freedom is won, the new ecclesia is born and he continues his missionary journeys. But the authorities soon catch up with the movement and Peter is imprisoned and chained: there is a violent earthquake and he is free. He shakes off his chains and finds his way to the house of friends. Full of joy they whisk him away to safety, and his prison guards are executed for allowing him to escape. (Plate X)

**Plate X—The Liberation of St. Peter—RAPHAEL (Vatican Museum)**

**Plate XI**

**Domine Quo Vadis?—CARACCI (National Gallery)**

**Plate XII—The Crucifixion of St. Peter—GUIDO REMI (Vatican Museum)**

**The Martyr**

There is a strong and fairly reliable tradition that after he gave up the leadership of the church in Jerusalem his missionary journey took him to Rome. The danger becomes even greater under Nero's persecution of the Christians and he is urged to leave the city. On the outskirts he has a vision, the Master

**Plate XIII**
**Detail of St. Peter from The Last Judgment—MICHELANGELO (Sistine Chapel)**

himself moving into the city carrying his cross — "Domine Quo Vadis?", and the answer, "to Rome to be crucified in your place". He turns round and re-enters the city to certain death. (Plate XI)

Tradition again says that he was caught and made to witness the crucifixion of his wife before he is finally nailed to a cross himself. Remembering his denial and the reverence in which he holds the Master he cannot feel worthy of even dying as He did, and so in utter humility the man who had become a rock was crucified upside down. (Plates XII, XIII, XIV)

Some of you may remember Dr. Carleton who used to like to hand to patients Beran Wolfe's "How to be happy though Human". In that book it is stated that "perfection is a curse and the cult of perfection, that is, living according to the motto of 'one hundred per-cent or nothing' restricts men and women to the narrowest spheres of isolation. Perfectionism is the

**Plate XIV**
**San Pedro—EL GRECO (The Escorial Museum)**

blinker that keeps many a man on the path of failur You will find the greatest souls among the mo modest men".

This is in many ways my conclusion tonight; th maturity is all the perfection we can reach, but it a maturity of the whole man and this includes t spirit of man and involves him in belief in and contact with the mystical and the miraculous, t "other dimension" of life.

Secondly, that maturity is only reached throu Humility, a characteristic but elusive virtue in Chr tianity, and perhaps in some other religions. If we t to cultivate it, we become self-conscious about whereas genuinely humble people are usually unawa of it.

With our perfectionist patients and with oursel it may be wise to lower our sights somewhat; f which of us does not overestimate his moral coura and strength? Our record is usually one of mixed su cess and failure.

If there *is* this living contact with a personal pow which can infuse a strength other than our own, th more people should know about it. For to keep a law is one thing but to love a person quite another, and within the reach of everyone. What a relief when the rule of love is substituted for the rule of law. The art of mature life seems to be to take the strain out of living without surrendering essential ideals; a loosening of restraints, a more liberal attitude to life.

This process is not without its dangers and many a highly principled man has plunged into licence as a result of this liberation. But if the humility is there and a faith in this other dimension, some kind of perfection may ultimately be reached, if by a more devious route.

*References*

Clark, Sir Kenneth. *Civilization,* B.B.C. Publications, 1969, p. 241.

Gollancz, Victor. *My Dear Timothy*, Gollancz, 1957.

Hadfield, J. A. *Psychology and Mental Health,* George Allen and Unwin, 1950, Chapter 11, p. 287.

Phillips, J. B. *Ring of Truth,* Hodder & Stoughton, 1967, p. 53.

Raverat, Gwen. *Period Peace,* Faber, 1952.

Reid, Charles. *Malcolm Sargent,* Hamish Hamilton, 1968, p. 396.

Reilly, Michael. *World Medicine,* February 24, 1971.

Schott, Rolf. *Michelangelo,* 1963, Thomas Hudson, pp. 110-111.

Wolfe, Beran. *How to be Happy though Human,* Routledge, p. 348.

## Figures and Tables

**Plate I f1:**
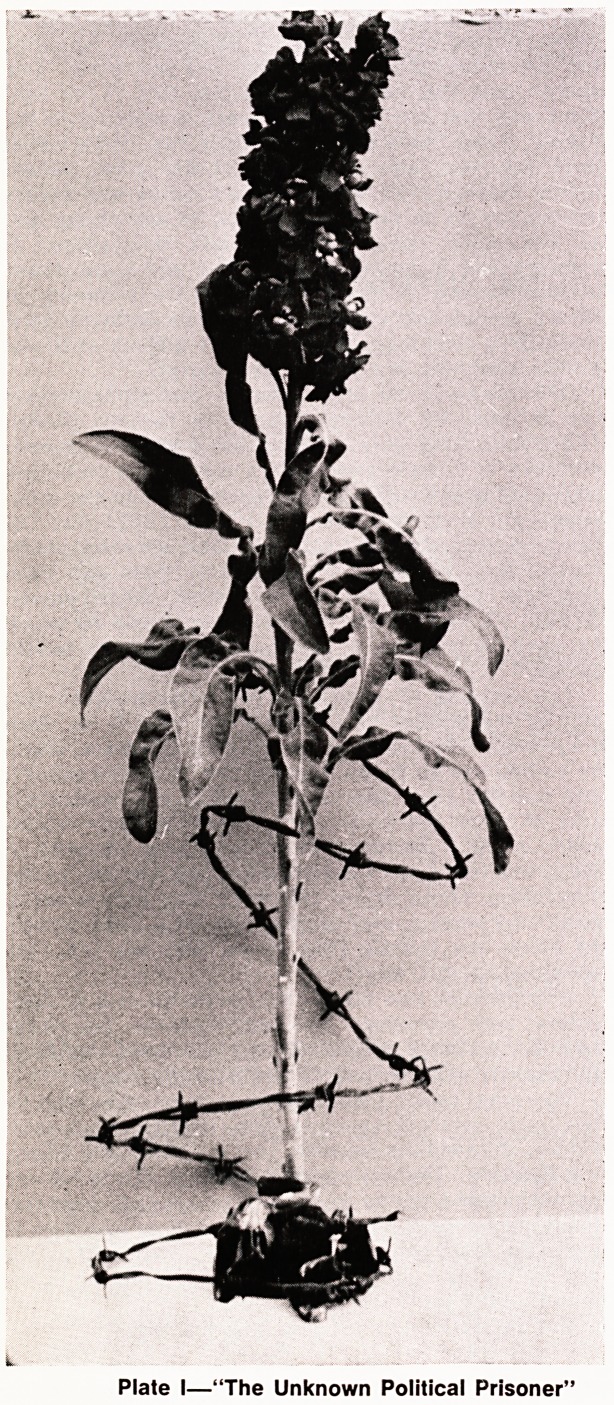


**Plate II f2:**
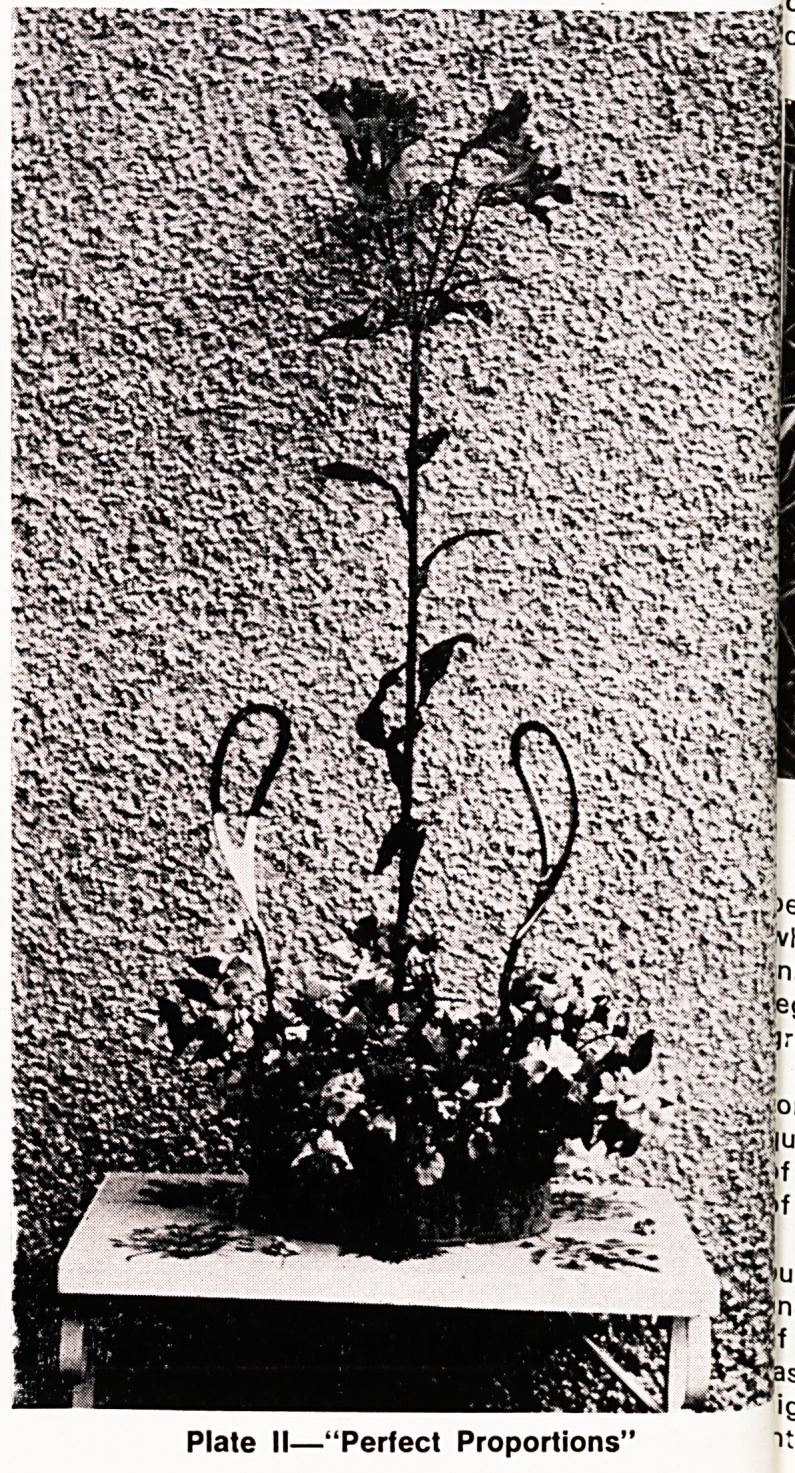


**Plate III f3:**
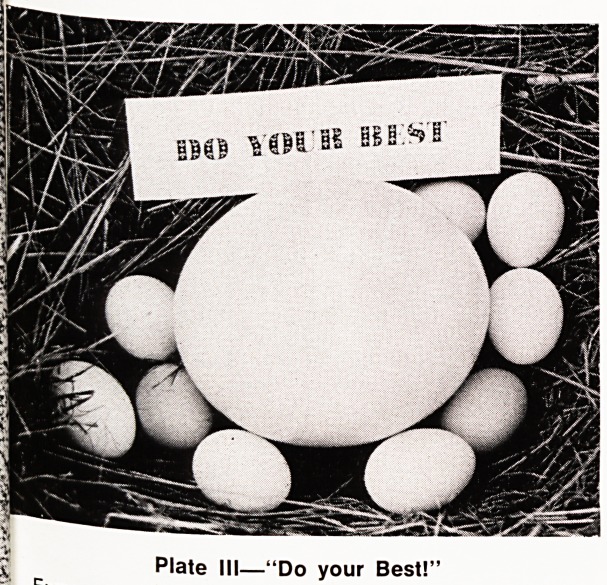


**Plate IV f4:**
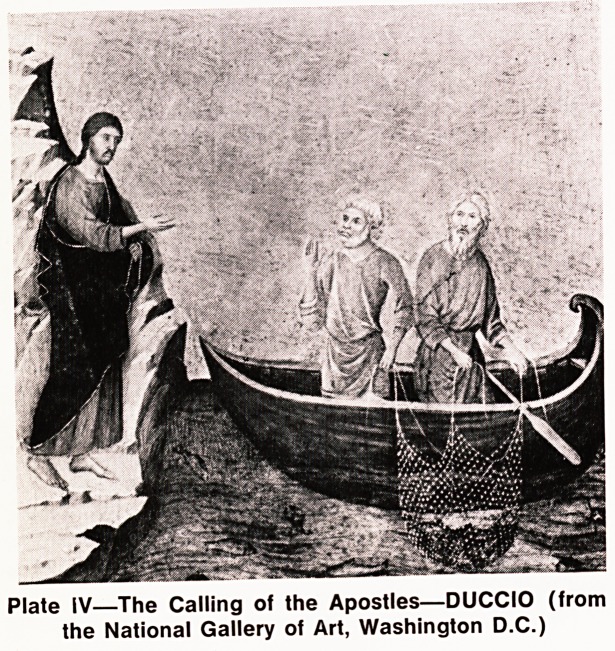


**Plate V f5:**
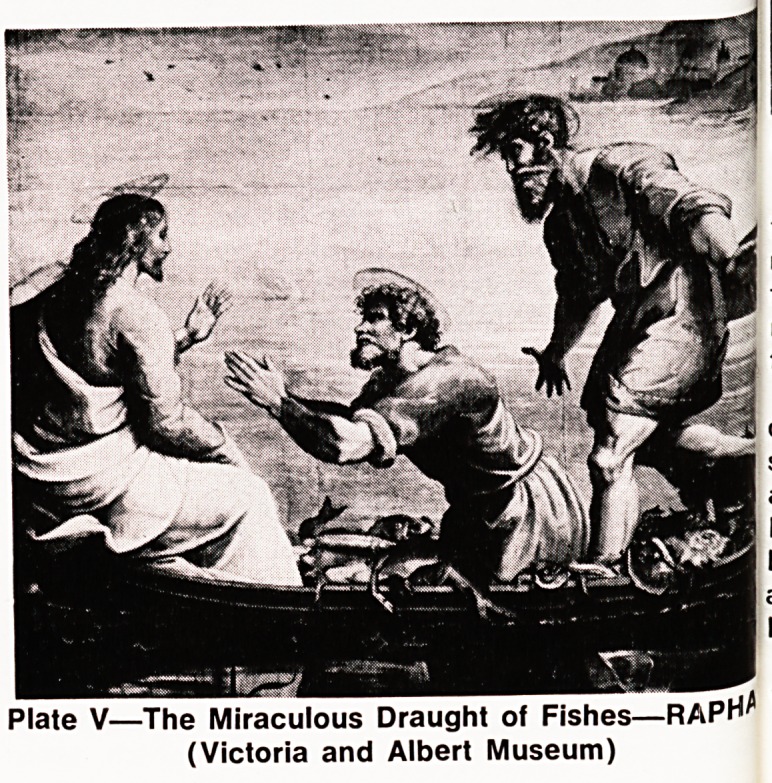


**Plate VI f6:**
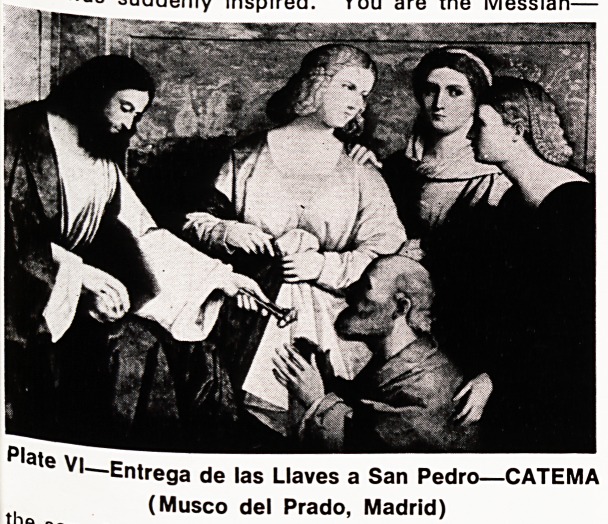


**Plate VII f7:**
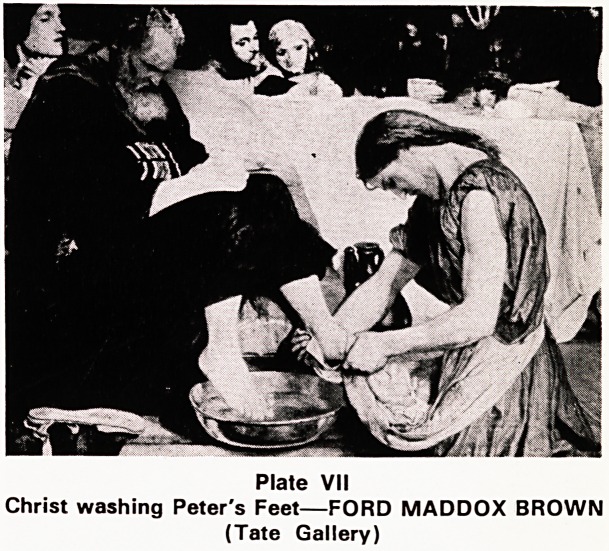


**Plate VIII f8:**
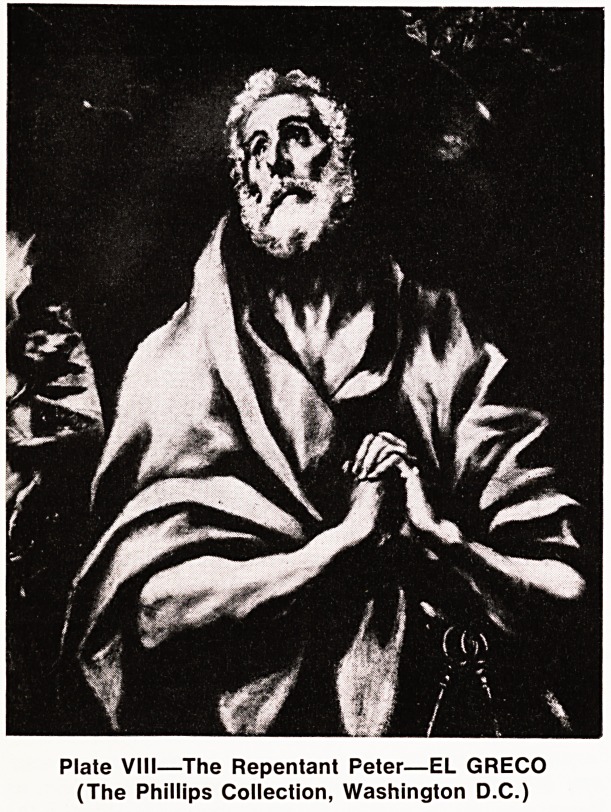


**Plate IX f9:**
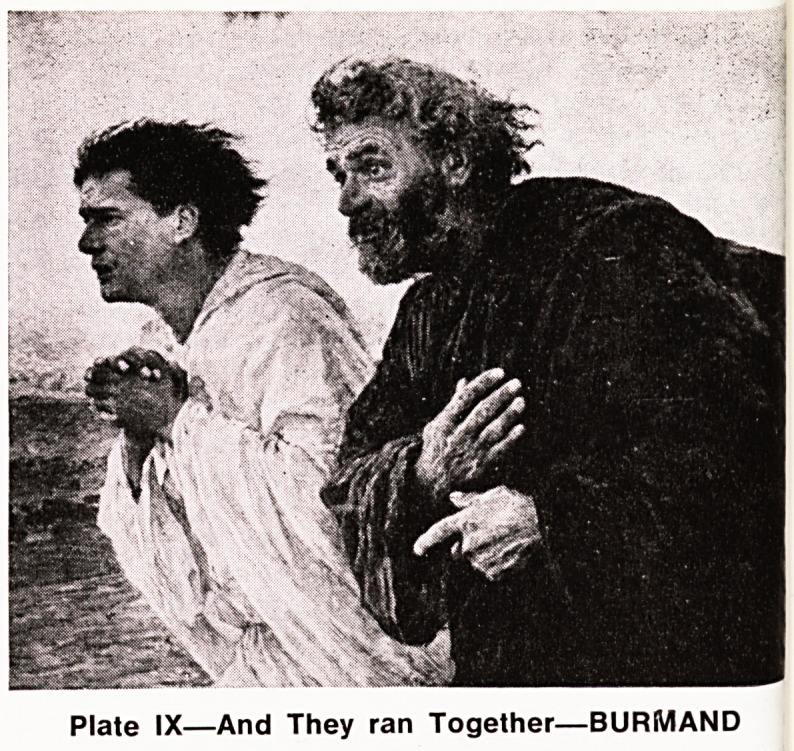


**Plate X f10:**
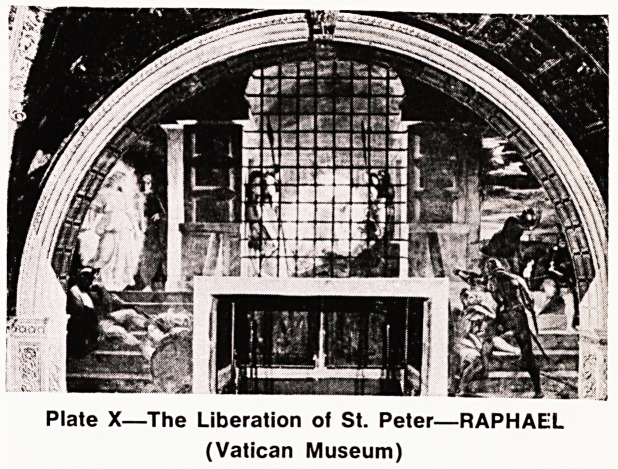


**Plate XI f11:**
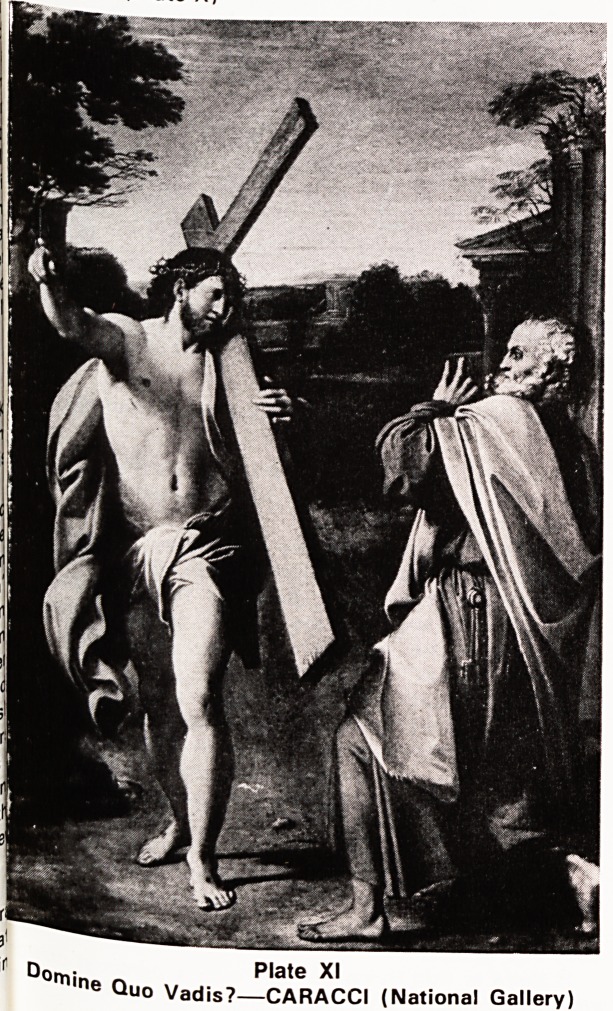


**Plate XII f12:**
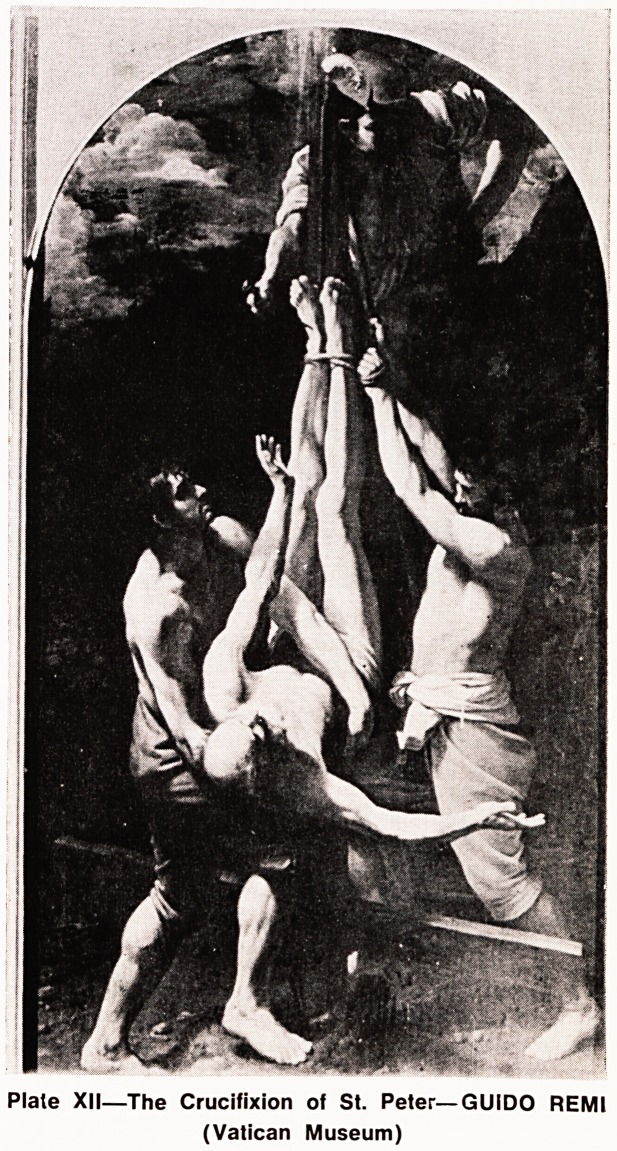


**Plate XIII f13:**
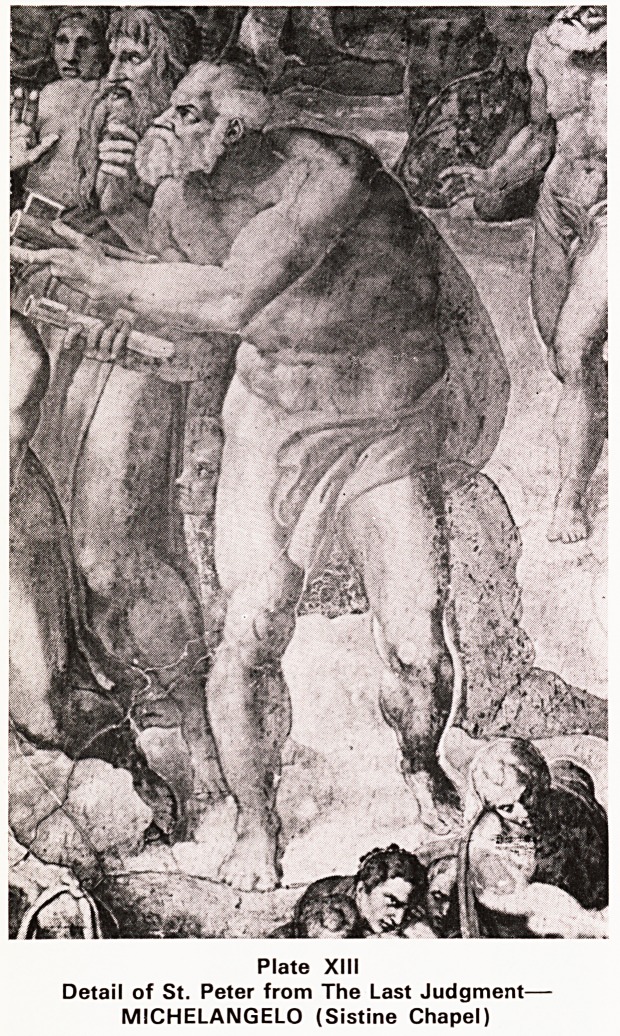


**Plate XIV f14:**